# Metabolic syndrome distributions in dietary diversity score groups and its associated factors among adults in the urban community of Jimma, Southwest Ethiopia: a community based cross- sectional study

**DOI:** 10.1186/s12902-022-01238-6

**Published:** 2022-12-22

**Authors:** Belay Zawdie, Temamen Tesfaye, Solomon Berhanu Moges, Yonas Tesfaye, Ayantu Kebede, Mulualem Tadesse, Esayas Kebede Gudina, Lelisa Sena Dadi, Dessalegn Tamiru, Tefera Belachew Lemma

**Affiliations:** 1 Department of Biomedical Sciences, Jimma University, Jimma, Ethiopia; 2 School of Nursing and Midwifery, Jimma University, Jimma, Ethiopia; 3 Departments of Epidemiology, Jimma University, Jimma, Ethiopia; 4 Department of Psychiatry, Jimma University, Jimma, Ethiopia; 5 Department of Medical Laboratory, Jimma University, Jimma, Ethiopia; 6 Department of Internal Medicine, Jimma University, Jimma, Ethiopia; 7 Department Nutrition and Dietetics, Jimma University, Jimma, Ethiopia

**Keywords:** Metabolic syndrome, Lipid profile, Dietary diversity, Ethiopia

## Abstract

**Background:**

Dietary diversity score has long been recognized as a key component of diets quality balances for healthy life status. However, diets with more variety of food items might increase calorie intake and body weight, which, in turn leads to central obesity (waist circumference).Therefore, this study aims to determine the prevalence of metabolic syndrome among dietary diversity score groups, and its associated factors among adults in the urban community of Jimma, Southwest Ethiopia.

**Methods:**

A total of 915 adults aged ≥ 18 years were randomly recruited in this cross-sectional study.The study was undertaken from June 17, 2019, up to July 27, 2019. To this end, the collected data were entered to Epi Data 3.1 and analysed using and SPSS 25 version. What’s more, a multivariable logistic regression was used to assess associated factors of the unrecognized metabolic syndrome; adjusted odds ratio (AOR) with its corresponding 95% CI, at *P-value* ≤ *0.05.*

**Results:**

The occurrence of metabolic syndrome was 14.4%, and it is more prevalent in females, 11.15% than males, and 3.25%. The most prevalent components of the metabolic syndrome were low level of high-density lipoprotein, elevated level of triacylglycerol, and waist circumferences. Even though metabolic syndrome is not significantly associated with any of the dietary diversity score groups, its prevalence distribution varies among the groups (6.6% in middle, 5.8% in high and 1.9% in low dietary diversity groups). With potential confounders adjusted, by 75% female was significantly associated with the occurrence of metabolic syndrome than male (102 vs. 29, AOR = 0.25 at 95%CI: 0.15–0.40, *P* = 0.001). Whereas, age ≥ 35 years old (104 vs. 27, AOR = 2.91 at 95%CI:1.78–4.86,*P* = 0.001), large family size > 5 (65 vs. 10,AOR = 2.43 95% CI: 1.10–5.36, *P* = 0.03), overweight and obesity (121 vs. 10, AOR = 6.97, 95% CI: 4.50 –10.83, *P* = 0.005), elevated total cholesterol (103 vs. 28,AOR = 2.46, 95% CI: 1.47–4.11, *P* = 0.001), and consuming ( spices, condemns and beverages) ≥ 4 days per week (79 vs. 52, AOR = 0.52, 95% CI:0.33 –0.82, *P* = 0.005) were positively associated with the prevalence of metabolic syndrome as compared to their counterparts.

**Conclusion:**

Unrecognized metabolic syndrome was relatively high in the study community. The prevalence of metabolic syndrome varied among dietary diversity groups. But any of the dietary diversity scoring categories was not significantly associated with the occurrence of metabolic syndrome. Thus, awareness needs to be made to practice healthy diet and regular physical activity to maintaining normal body weight. Moreover, early screening of metabolic syndrome should be promoted.

## Background

Even though there is an ongoing international debate on MetS’s definitions and existence, there are many official definitions of it [1, 2]. For the current study purpose, the authors used the definition of International Diabetic Federation (IDF). According to the new IDF definition, MetS is defined as central obesity (defined by waist circumference) plus any two of the risk factors: raised triglyceride, low high-density lipoprotein (HDL) cholesterol, raised blood pressure, and/or raised fasting plasma glucose level [3]. Since, MetS is the result of the highly complex interplay among several risk factors; a single etiology cannot be assigned to define it [1, 2]. For this reason, MetS is defined by various organizations and scholars, the IDF [3], Executive Summary of the Third Report of the national cholesterol Education program(NCEP) expert panel discussion [4], and World health organization [5–7].

Based on the IDF criteria, up to 25% of the world’s adults have MetS [3]. This is particularly common on adults who are lower levels of obesity in Asians compared to Europeans. After considering the difficulties in recognizing pooled criteria that are relevant through all ethnicities for MetS, the IDF suggests as using a new set of criteria per ethnic/racial specific cut-offs. Moreover, the IDF also recommends using European criteria, for where there are limited data in sub-Saharan Africa countries [3].

Furthermore, MetS is a metabolic derangement associated with the increased risk of Cardiovascular Diseases (CVDs) and Diabetes Mellitus (DM). Likewise, MetS is characterized by a special pattern of reversible major risk factor for Type 2 Diabetes Mellitus (T_2_DM) and CVDs. Plus, to this, MetS is not a disease by itself; it is a set of undesirable condition rooted in one’s poor lifestyle. It is also associated with the increased prevalence of obesity [1, 3, 8].

On top of this, MetS is a group of clinical and biological abnormalities with a global prevalence of 20–25% in the adult population [9]. According to a joint interim statement,in Japan, MetS’s prevalence has been reported to range from 18.5% to 37.3% in men and 4.4% to 12.8% in women [10, 11]. Besides, the magnitude of MetS among T_2_DM patients in Tigray, North Ethiopia was 51.5% and it’s associated with overweight, and obesity has been increasing in sub-Saharan African countries [12, 13].

According to the WHO, Chronic Non-communicable Diseases (NCDs) related to obesity will exceed that of infectious diseases in Sub-Saharan Africa by 2030 [14, 15]. Among NCDs, MetS is the commonest consequence of malnutrition [16]. In recent years, economic development and urbanization have rapidly changed the dietary habits shifts to processed food, which raise great concern about obesity and other health associated outcomes [17].

However, a study conducted in Southwest China showed that people with medium and high DDS were at higher risk of general and central obesity than people with less DDS. According to this study high DDS was associated with excessive energy intake calories induced obesity. In line with this, public health messages about DDS emphasis only on widely recommended for health with less stress [18]. On the contrary, there is study showed that higher DDS had an inverse association with MetS and some of its features. Therefore, a higher DDS might be associated with a lower possibility of having some metabolic disorders [19].

A further point is that earlier studies have documented adult lifestyles like smoking cigarette, drinking alcohol, less physical exercise, and consuming unhealthy dietary habits as the risk factors for the development of MetS [20–22]. But, there are limited data on the occurrence of MetS and its risk factors among apparently healthy communities in Ethiopia. Therefore, this study attempted to detect the distribution of unrecognized MetS varied in DDS groups and its associated factors among adults in the urban community of Jimma, southwest Ethiopia.

## Materials and methods

### Study setting and period

Urban community-based cross-sectional study was conducted among adults aged greater or equal to 18 years. Data were collected from June 17 to July 27, 2019. It was conducted in Jimma town, Southwest Ethiopia.

### Population, sample size and sampling technique

All adults who have been living in Jimma town for at least six months before data collection randomly recruited for the purpose of this study. The sample size was determined using the single population proportion formula considering 17.9% of prevalence of MetS in Addis Ababa, Ethiopia [22], 95% confidence interval (CI), 3% margin of error, 10% non-response rate, and 1.5 design effect.

$${N=\left(\frac{Z\alpha }{2}\right)}^{2}P\left(\frac{1-P}{{d}^{2}}\right)=\frac{{\left(1.96\right)}^{2}(0.179x0.821)}{{0.03}^{2}}=\frac{3.8416x0.144375}{0.0009}= \frac{0.56555}{0.0009}=628$$

10% of non-respondents rate = 62.8

1.5 design effect of 628 = 942

Final sample size = 1005.

A multi-stage sampling technique was employed, where from 17 Kebeles (the lowest administrative structure in Ethiopia) in the town; six Kebeles were purposively selected by considering the settlement cluster, as the district sampling unit. Then, the sample size number of adults was allocated into the selected Kebeles by considering population proportion to the total number of households in each Kebeles. Then, households in each Kebele were selected by systematic sampling technique and the lottery method was used to select the study participants where more than one eligible adult have been living in the same household.

### Data tool development and collection techniques

Data were collected using WHO STEPS questionnaire [23] adapted to the local context based on the study objective. The questionnaire was first adopted and written in English, translated into Amharic and Afaan-Oromo by experts, and then translated back into English by a panel of professionals. A four-day training of the contents of the questionnaire, data collection techniques, and ethical concern of human research was provided to the research interviewers prior to the commencement of the study. The questionnaire was pretested in Agaro town of Jimma zone which has similar characteristics with the study area before the initiation of the study, to check the flow of the questionnaires, measure the length of time required for interviews, feasibility, and the clarity of the language used. Data were collected by six trained BSc nurses, along with six health extension workers permanently works at each kebeles.,. All interviews and measurements were done with close supervision of the research team.

### Socio-demographic and behavioral characteristics

Socio-demographic and behavioral characteristics were collected through face-to-face detail interviews using open-ended questionnaires. WHO guideline-recommended on physical activities as all adults should undertake 150–300 min of moderate-intensity, or 75–150 min of vigorous-intensity physical activity, per week. Therefore, as if the study participant’s physical activity trend fitting with the WHO recommendation categorized as physically active and did not fit categorized as physically inactive. Similarly, the sedentary behavior of the study participants was measured by interviewing about the time spent sitting during a typical week and their responses were classified as categories of sedentary (< 4 h/day) and not sedentary (≥ 4 h/day) [24]. Likewise, khat chewing and tobacco using behavior were defined as self-reported currently chewing of khat and practicing of smoked tobacco or smokeless tobacco products, respectively. Current alcohol users are defined as people who drink alcohol more than one day per week [25].

### Anthropometric measurements

In this step, height, weight, waist, and hip are needed to calculate body mass index (BMI), waist circumference (WC), waist to hip ratio. Therefore, the height of the study participants was measured to the nearest 0.1 cm using an adjustable portable stadiometer with the subjects positioned at the Frankfurt Plane, the four points (Heel, Calf, Buttocks, and Shoulder) touching the vertical stand and their shoes were taken off. Before starting the measurements, the stadiometer was checked using calibration rods. Likewise, Weight was measured with a digital scale (Model 871, Seca, Germany) accurate to 100 g with the subjects wearing very light clothes and shoes taken off. The validity of the scale was checked using an object of a known weight every morning and between the measurements. After BMI was calculated by weight (kg)/height (meter)^2^ performed, BMI of the study participants was categorized as underweight (< 18.5 kg/m^2^), normal [18.5–24.9 kg/m^2^], overweight [25–29.9 kg/m^2^] and obese (≥ 30 kg/m^2^) [26, 27]. Similarly, WC was measured at the middle between the lowest costal margin at the mid-clavicular line and the anterior superior iliac spine using a fixed tension tape meter without any pressure to the body surface and then measurements were recorded to the nearest 0.1 cm. The waist-to-hip ratio was calculated as WC divided by hip circumference. According to the WHO recommendation, WC values > 94 cm for men and > 80 cm for women were considered as high [28]. Therefore, as per the WHO, the study participants were grouped as high and low WC.

### Blood pressure measurement

Blood pressure (BP) was measured in a sitting position from the right arm. It was measured in triplicate using an Aneroid Sphygmomanometer with small, medium, and large handcuff size [29], as fit to the subjects take rest for a minimum of five minutes. Then, two more successive measurements within five minutes apart were done. As per the WHO recommendation, the mean of systolic and diastolic blood pressures was considered for analysis. Accordingly, participants were considered as prehypertensive as if systolic BP was 120–139 and diastolic BP 80–89 mmHg, and hypertensive as if systolic BP ≥ 140 mmHg and/or diastolic BP ≥ 90 mmHg [30].

### Biochemical measurements

A five Milliliter venous blood sample was taken in a sitting position according to the standard protocol from each study participant and centrifuged within 30–45 min of collection. The blood Sample was used to determine either random or fasting blood glucose (12–14 h fasting) levels or lipid profiles (HDL, LDL, TG, and TC). Blood glucose was measured by using a glucometer soon sample drowns and measured in the laboratory too. Blood serum was carried out in ABX Pentra 400 Automated Chemistry Machine (Horiba ABX SAS, 34,184 Montpellier, France) at Jimma Medical Center (JMC) Clinical chemistry core laboratory to determine serum lipid profiles and glucose. But Low-Density Lipoprotein(LDL) level was calculated by using the Freidwald formula [31]. After analysis, TC ≥ 200 mg/dl and < 200 mg/dl were considered as high and normal respectively. Likewise, LDL was considered as optimal when LDL < 100 mg/dl but elevated as LDL ≥ 100 mg/dl. Similarly, TG level was considered as normal when TG < 150 mg/dl and HDL was considered as desirable/normal if it is > 40 mg/dl for men and > 50 mg/dl for women, otherwise it was categorized as low.

Based on the American Diabetes Association (ADA), Diabetes Mellitus (DM) is considered as positive (diabetic) with criteria of fasting blood glucose of ≥ 126 mg/dl and/or random blood glucose of ≥ 200 mg/dl. But considered as pre-diabetic with criteria of RBG: 100 mg/dl ≤ FBG ≤ 126 mg/dl and RBG: 140 mg/dl ≤ RBG < 199 mg/dl [32, 3].

### Metabolic syndrome measurement

Following the IDF criteria, study subjects were classified as MetS if participants had central obesity (defined as a waist circumference of ≥ 95 cm for men and ≥ 80 cm women plus two of any of the following risk factors: (1) raised TG level (≥ 150 mg/dL) or specific treatment for this lipid abnormality; (2) reduced HDL-C(< 40 mg/dL in males and < 50 mg/dL in females) or specific treatment for this lipid abnormality; (3) raised blood pressure (systolic BP ≥ 130 or diastolic BP ≥ 85 mmHg) or treatment of previously diagnosed hypertension; (4) raised FBG (≥ 100 mg/dL) or previously diagnosed with type 2 diabetes [7], and study subjects categorize as normal if participants had either normal central obesity or any two of the two risk factors values in the normal range [3].

Unrecognized metabolic syndrome in this study supposed to be operationalized as the study participants existing in community without confirmed positive MetS and its components before this study.

### Dietary diversity score and survey

Dietary diversity score was based on 24-h recall of the study participants consumption of food groups within the past 24-h using the Dietary individual or Women Dietary diversity Guidelines [33].

Trained interviewers from Jimma Medical Center (JMC) paired with local health extension workers visited the selected adult/s from the selected households to collect and register the information on food consumption using the 24-h dietary recalls.

Based on the guideline, all food items (16) were categorized into 9 groups which were grains (including cereals, tubers, and roots), vegetables, fruits, meat (including pork, beef, poultry, and organs), beans (including beans, nuts, and seeds), eggs, fish (including seafood, freshwater fish and aquatic products), dairy (including milk and products), and oil (including animal and vegetable oil). If a participant consumed any food item from any of the above-mentioned categories, he/she would get one point in that food category. If not, he/she would be scored zero. Consuming different foods from the same category would assume not count repeatedly. The total score would be the sum scores of the nine food groups and the maximum score could go up to 9. Therefore, for this study purpose, the DDS was categorized into low (1-3), medium (1-3), and high (1-3), [34].

### Data processing and analysis

The data were checked for completeness, coded and entered into EPI data 3.1, and exported to SPSS 25 version for the analysis. Descriptive analysis was explored using frequency and percentage for categorical variables. Continuous variables were expressed as mean ± SD. Normality was checked for continuous variables and transformation was made for data that are not normally distributed. Bivariate and multivariate logistic regression analysis was used to evaluate the differences in the distribution of categorical variables for the study groups. *P-value* < 0.2 was used as a cut-off to include variables for the multivariate binary logistic regression model. The result of the OR was used for interpretation of the strength of prediction of the independent variables to the dependent outcome. For all statistical significance tests, the cut-off value set was *P* < *0.05* with CI of 95%.

## Results

### Participants’ socio-demographic and behavioral characteristics

A total of 915 adults aged ≥ 18 years old were participated in the study. Nearly half, 439 (48%), of the respondents were females. The mean age of the respondents was 38.4 ± 13.5 years whereas the minimum and maximum ages were 19 and 88 years, respectively. Among the total study participants, 72 (7.9%), 153 (16.7%) and 321(35.1%), have been currently smoking cigarettes, drinking alcohol, and chewing khat, respectively. Around 604(66.0%) of the study participants did not practice physical exercises as per WHO recommended and 531(58.0%) represented under sedentary life. Nearly, most of the study participants, 722(78.9%) often have been using vegetable oil in meal preparation. These socio-demographic and behavioral Characteristic of the respondents are presented in (Table 1).

**Table 1:** Socio-Demographic and Behavioral Characteristic among Adults in Urban Community of Jimma, Southwest Ethiopia 2019 (N = 915)

**Variable**	**Category**	**Frequency**	**Percent**
Sex	Female Male	439 476	47.9 52.1
Age group(yrs)	<35 ≥35	403 512	44.0 56.0
Marital status	Single Married Divorced Widowed	30 654 131 100	14.2 71.5 14.3 10.0
Occupation	Unemployed Employed Private work	316 316 283	34.5 34.5 31.0
Family size	1–2 3–5 >5	151 483 281	16.5 52.8 30.7
Annual income (Ethiopia Birr)	≤10,000 10,001–49,999 ≥50,000	374 446 95	40.9 48.7 10.4
Currently Smoking	Yes	72	7.9
	No	843	92.1
Currently drinking alcohol	Yes	153	16.7
	No	762	83.3
Currently khat chewing	Yes	321	35.1
	No	594	64.9
Type of oil or fat used in meal preparation	Vegetable oil Butter, margarine	722 193	78.9 21.1
Level of physical activity	Insufficient	604	66.0
	Sufficient	311	34.0
Sedentary life	<4 hours ≥4 hours	384 531	42.0 58.0

### Anthropometric and biochemical measurements

More than one-fourth, 255(27.8%), of the participants have been overweight and obese. And 194(21.2%) of the respondents have been existing with confirmed diagnosed hypertension for this study purpose. On the other hand, 672(73.4%) had a high west-to hip ratio and 126 (13.8%) of the participants have had more than 200 mg/dl total cholesterol. In this study, Mets prevalence was 131(14.3%) with a higher prevalence in females, 102 (11.14%), than males. Moreover, low HDL 455 (49.7%), elevated LDL 311(34.0%), elevated TAG 255(27.9%) and Central obesity 217 (23.7%) were the high to low prevalent component of the MetS (Table 2).

**Table 2:** Physical and Biochemical Measurements Characteristic among Adults in Urban Community of Jimma, Southwest Ethiopia 2019 (N = 915)

**Variable**	**Category**	**Frequency**	**Percent**
Anthropometry indices	Body mass index Underweight Normal Overweight Obese		
		135	14.8
		525	57.4
		177	19.3
		78	8.5
	Waist circumferences Normal Central obesity	698	76.3
		217	23.7
	Waist to hip ratio		
	Normal	243	26.6
	Elevated	672	73.4
Hypertension	Yes	194	21.2
	No	721	78.8
Lipid profiles	Total cholesterol <200 mg/dl ≥200 mg/dl		
		789	86.2
		126	13.8
	HDL cholesterol Low Normal		
		455 460	49.7 50.3
	LDL cholesterol <100 mg/dl ≥100 mg/dl		
		604	66.0
		311	34.0
	Triacylglycerol <150 mg/dl ≥150 mg/dl	660	72.1
		255	27.9

### Metabolic syndrome (MetS) and dietary diversity score (DDS)

**Fig. 1:**
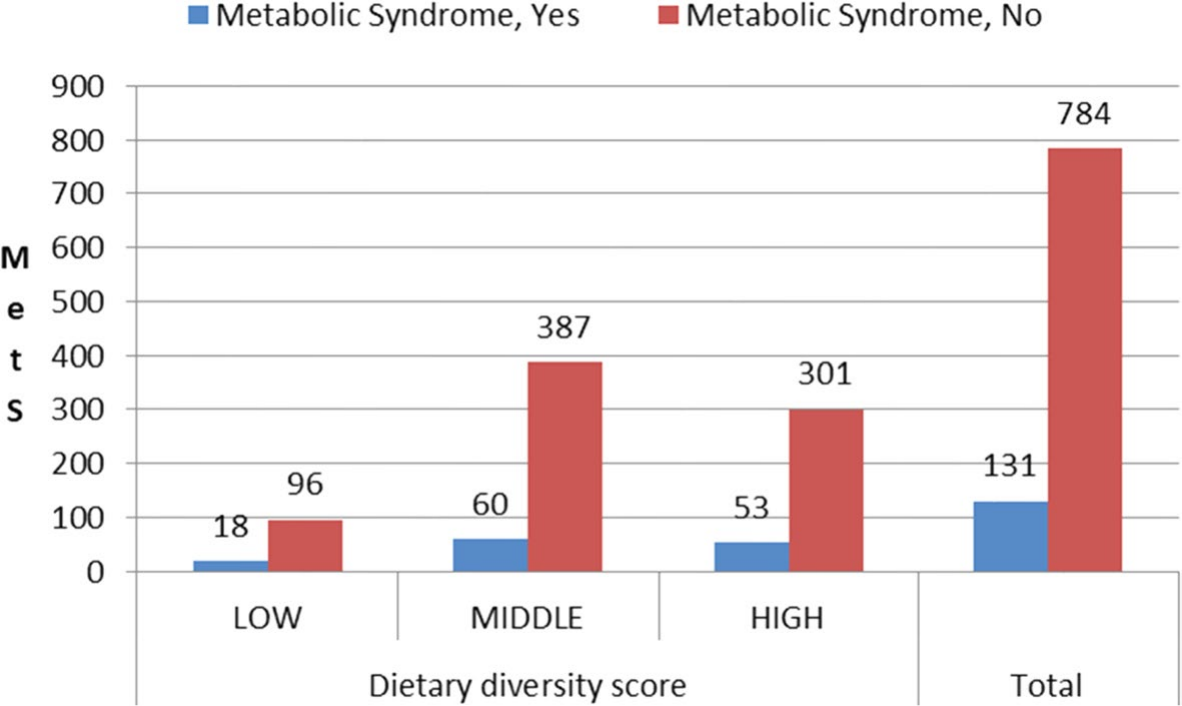
Metabolic Syndrome occurrences among DDS Group of Adults in Urban Community of Jimma, Southwest Ethiopia 2019(*N* = 915)

The frequency and percentage distribution of low, middle and high DDScatagories were represented by 114(12.5%), 447(48.8%) and (35,436.6%) respectively. The prevalance of the MetS remarkaibly varied among DDSgroups. Its prevalance was 60(6.6%) in middle, 53(5.8%) in high and [18] 1.9% in low DDS groups.The prevalence of MetS among the DDScategories patter is the same with non-MetS (Fig. 1).

### Factors associated with metabolic syndrome

Age, sex, occupation, level of physical activity, sedentary life, type of oil used for cooking, middle DDS,elevated cholesterol, large family size,current smoking of cigarette, current drink of alcohol, consuming (spices,condemns and beverages) more than 4 days per week, and BMI were included in multiple logistic regression for having *P*-value < 0.2. In multiple logistic regression only age > 35 years old, female, family size > 5, consuming (spices, condemns and beverages) more than 4 days per week, overweight and obese BMI and elevated cholesterol were significantly associated with metabolic syndrome at *P*-value < 0.05.

In the final model, by 75% female was significantly associated with the occurrence of metabolic syndrome than male (102 vs. 29, AOR = 0.25 at 95%CI: 0.15–0.40, *P* = 0.001). Whereas, age ≥ 35 years old (104 vs. 27, AOR = 2.91 at 95%CI:1.78–4.86, *P* = 0.001), large family size > 5 (65 vs. 10,AOR = 2.43 95% CI: 1.10 –5.36, *P* = 0.03), overweight and obesity (121 vs. 10, AOR = 6.97, 95% CI: 4.50 –10.83, *P* = 0.005), elevated total cholesterol (103 vs. 28,AOR = 2.46, 95% CI: 1.47–4.11, *P* = 0.001), and consuming ( spices, condemns and beverages) more than 4 days per week (79 vs. 52, AOR = 0.52, 95% CI:0.33 –0.82, *P* = 0.005) were positively associated with the prevalence of metabolic syndrome as compared to their counterparts (Table 3).

**Table 3:** Bivariate Regression Analysis to Identity Risk Factors Associated with Unrecognized MetS among Adults in Urban Community of Jimma, Southwest Ethiopia 2019(N = 915)

**Variables**	**Metabolic syndrome**		**COR,95%CI**	**AOR,95%CI**
	**Yes**	**No**		
Sex of the participants				
Female Male	102	337	1	1
	29	447	0.21 (0.14–0.33)	0.25(0.15–0.40)**
Age				
< 35 years ≥ 35 years	27	376	1	1
	104	408	3.55(2.27–5.54)	2.9(1.78–4.86)**
Family size				
(1, 2)	10	141	1	1
(3-5)	56	427	2.193(1.10–4.38)	1.91(0.88–4.10)
**(**> 5)	65	216	3.51(1.73–7.10)	2.43(1.10–5.36) *
Using (Spices, condiments, beverages)				
No	52	260	1	1
Yes	79	574	0.556(0.38–0.82)	0.52(0.33–0.82)**
BMI				
≤ 24.99 kg/m^2^	10	650	1	1
≥ 25 kg/m^2^	121	134	8.60(5.71–12.96)	6.97(4.50–10.83)**
Total cholesterol				
< 200 mg/dl	28	761	1	1
≥ 200 mg/dl	103	23	3.23(2.07–5.01)	2.46(1.47–4.11)**

^**^P = 0.001; ^*^P = 0.05; BMI Body mass Index

## Discussion

Nowadays, the prevalence of MetS has increased worldwide and it is considered as a major public health problem [35–37]. Hence, this study attempted to detect the magnitude of MetS and its variation with the DDS among adults in the urban community of Jimma, Southwest Ethiopia.

The main finding of this study indicates that MetS and its associated factors is existing as a major health concern of adults who were unaware of the disorder in the study community. In the current study, the prevalance of the MetS remarkably varied among DDS groups. Even though larger prevalence of the MetS has been observed in middle and higher DDS as compared with low DDS, the association is not statically significant. In addition to that, the pattern of variations of MetS as compared with non-MetS among DDS catagories near similar (Fig. 1). Contrary to this, other studies revealed as the high dietary diversity intake is favorably associated with MetS [18, 34, and 38] and it’s inversely associated with MetS and some of its features [19].

Finally, sex (female), advancing age, participants who have been consuming (spices, condemns and beverages) more than 4 days per week, having larger family size, hypercholesterolemia, being overweight and obese were independently increase the odds of metabolic syndrome (Table 3).

What more, the magnitude of MetS among the study participants was 14.3% which is near to the finding obtained from working adults in Addis Ababa, Ethiopia (17.9%) [22], also the result lies within the range of 12–86 from sub-Saharan Africa study [12]. Indeed, it is higher than on the result found from residents of Mizan-Aman town of southwest Ethiopia (9.6%.) and NCDs in Ethiopia (4.4%) [39, 40]. But, the current result indicated low prevalent rate as compared with the study conducted among adult urban dwellers of northern Ethiopia (21.8%). This is more verified by results from systematic review and meta-analysis on Ethiopia population (27.92%), on type T_2_DM patients in Ethiopia (57%), on adults of across European countries (24%) and UK (32%), people with T_2_DM of Gahanna (68.6%), Nigerians with type T_2_DM (68.7%) and on T_2_DM patients in Kerman of Iran (64.9%) [41–47]. This difference is due to differences in study subjects, setting, sample size, methods, socio-economic status, ethnicity, lifestyle, cut-off differences, the criteria used to define MetS, and other contributing factors for the higher province of MetS.

A further point is that, the odd of MetS three times higher in adults whose age was greater than or equal to 35 years than less. This may be due to age-related physiological processes and environmental factors altered. For example, gradual decrease in the basal metabolic rate, dyslipidemia, stress-induced hypercortisolism, hypogonadism, decreased growth hormone secretion, concomitant insulin resistance, and abdominal fat deposition, changes of the functioning of beta cells and other environmental and physiological factors, may trigger a genetic expression of MetS which becomes more prominent with biological maturation [48, 49].

By the same token, the prevalence of MetS was higher in females than males. The result of the higher prevalence of MetS among aged and female participants agreed with previous other related studies [9, 12, 23, 46, 47]. Higher MetS in females than males might be due to the use of hormonal oral contraceptives that can decrease insulin sensitivity, glucose tolerance, increase blood pressure and increase weight gain; menopause promotes a change in body fat distribution to increase central adiposity [50]. Additionally, less proportion of females were involved in regular physical exercise than males in this study, which might have its own contribution to the observed higher MetS prevalence among them. Others justification is that in women; low HDL cholesterol, elevated BMI, increased waist circumference and hyperglycemia were significantly larger contributors to the MetS as compared with male [51].

In the current study, the odd of MetS was seven times higher among participants', BMI ≥ of 25 kg/m^2^(include overweight and obese) as compared to those, BMI ≤ of 24.99 kg/m2) (include normal and underweight). This is higher with studies done in Ethiopia, Gahanna, Nigeria, and Iran [42, 44–46]. This is due to obesity which rises in countries like Ethiopia experiencing domestic nutrition transition from a primarily plant-based diet to meat and processed food diet associated with weight gain and chronic illnesses and the types of body fat distribution are still the core aspects of insulin resistance which links to MetS. Furthermore, in the current study, for participants with hypercholesterolemia (≥ 200 mg/dl), the odd of MetS was 2.46 times higher than the normal one. This finding is in line with the higher cholesterol synthesis and cholesterol excretion as neutral sterols in patients with MetS than in healthy populations [52].

In general, the findings of the present study showed that MetS is major prevalent with its risk factors in the community among adults in Ethiopia. Early identification of MetS and its risk factors among adults is of great importance since MetS suggest increased risk of morbidities such as CVD, decreased quality of life, increased health care cost, as well as mortality. Therefore, NCDs treating center must strengthen appropriate and targeted prevention strategies such as encouraging people to adopt dietary modification and physical activity which are reported to reduce incidence and progression of MetS [53]. Moreover, there should be a more frequent screening of adults in the community for MetS components and risk factors prior to full blow development of MetS.

The Strengths of this study include appraising the prevalence of MetS occurrences varied under DDS groups and its predictors at the community level and providing a baseline for future studies to early monitor MetS and risk factors as well as in health planning and managements strategies. This stud has also the limitations relating to the cross-sectional study design of which provides a snapshot of the burden at a particular moment in time. Additionally, the study also designed to include only adults who were ≥ 18 years which it was not included all age groups and epidemiological representing areas; therefore, it’s miss national representative. Since DDS is not quantified to calories and nutrient contents, it is not in concluding position to indicate the causes and effect relationship with MetS, It is also limited to show the health-related outcome in long term impacts and economic consequences of MetS among adults living in the community in Ethiopia.

## Conclusion

Unrecognized MetS and its components were prevalent among adults in the study community. Moreover, sex (female), advancing age, participants who have been consuming (spices, condemns and beverages) ≥ 4 days per week, having larger family size, hypercholesterolemia, being overweight and obese were independently increase the odds of MetS. Even if higher occurrence of MetS in middle and high DDS as compared with low DDS categories, their association was statistically not significant. Thus, awareness needs to be created among the community to practice regular physical activity and maintaining normal body weight. Additionally, screening of MetS should be promoted for early detection, prevention, and treatment.

## Data Availability

The data sets generated and or analysed during the current study are not publicly available due to personal data protection legislation but are available from the corresponding author on reasonable request.

## Abbreviations

*MetS*: Metabolic Syndrome
*DDS*: Dietary Diversity Score
*NCEP ATP III*: National Cholesterol Education Program’s Adult Treatment Panel III
*AOR*: Adjusted Odds Ratio
*BMI*: Body Mass Index
*BP*: Blood Pressure
*CI*: Confidence Interval
*COR*: Crude Odds Ratio
*DM*: Diabetes Mellitus
*T*_*2*_*DM*: Type 2 Diabetes Mellitus
*FBS*: Fasting Blood Sugar
*HDL*: High Density Lipoprotein
*IDF*: International Diabetes Federation
*JMC*: Jimma Medical Center
*LDL*: Low-Density Lipoprotein
*NCD*: Non-Communicable Diseases
*RBS*: Random Blood Sugar
*TC*: Total Cholesterol
*TAG*: Triacylglycerol
*WC*: Waist Circumference
*WHO*: World Health Organization
